# Bedrock-alluvial streams with knickpoint and plunge pool that migrate upstream with permanent form

**DOI:** 10.1038/s41598-019-42389-2

**Published:** 2019-04-16

**Authors:** Li Zhang, Toshiki Iwasaki, Tiejian Li, Xudong Fu, Guangqian Wang, Gary Parker

**Affiliations:** 1 Department of Civil & Environmental Engineering, University of Illinois Urbana-Champaign, Urbana, 61801 USA; 2 State Key Laboratory of Hydroscience and Engineering, Tsinghua University, Beijing, 100086 China; 3 School of Water Resources and Electric Power, Qinghai University, Xining, 810016 China; 4 Civil Engineering Research Institute for Cold Regions, Sapporo, 062-8602 Japan; 5 Department of Geology, University of Illinois Urbana-Champaign, Urbana, 61801 USA

## Abstract

Purely alluvial rivers cannot sustain knickpoints along their long profiles, as they would be obliterated by diffusional morphodynamics. Bedrock streams with a partial alluvial cover, however, form and sustain slope breaks over long periods of time. Here we consider the case of an initial profile of a bedrock-alluvial stream with a sharp slope break, or knickpoint, from high to low midway. We show that if the initial flow is sufficiently Froude-supercritical in the upstream reach and Froude-subcritical in the downstream reach, a three-tiered structure can evolve at the slope break: a hydraulic jump at the water surface; a scour hole in the alluvium above the bedrock, and a plunge pool carved into bedrock. Once the profile adjusts to balance uplift, it can migrate upstream without changing form.

## Introduction

**Figure 1:**
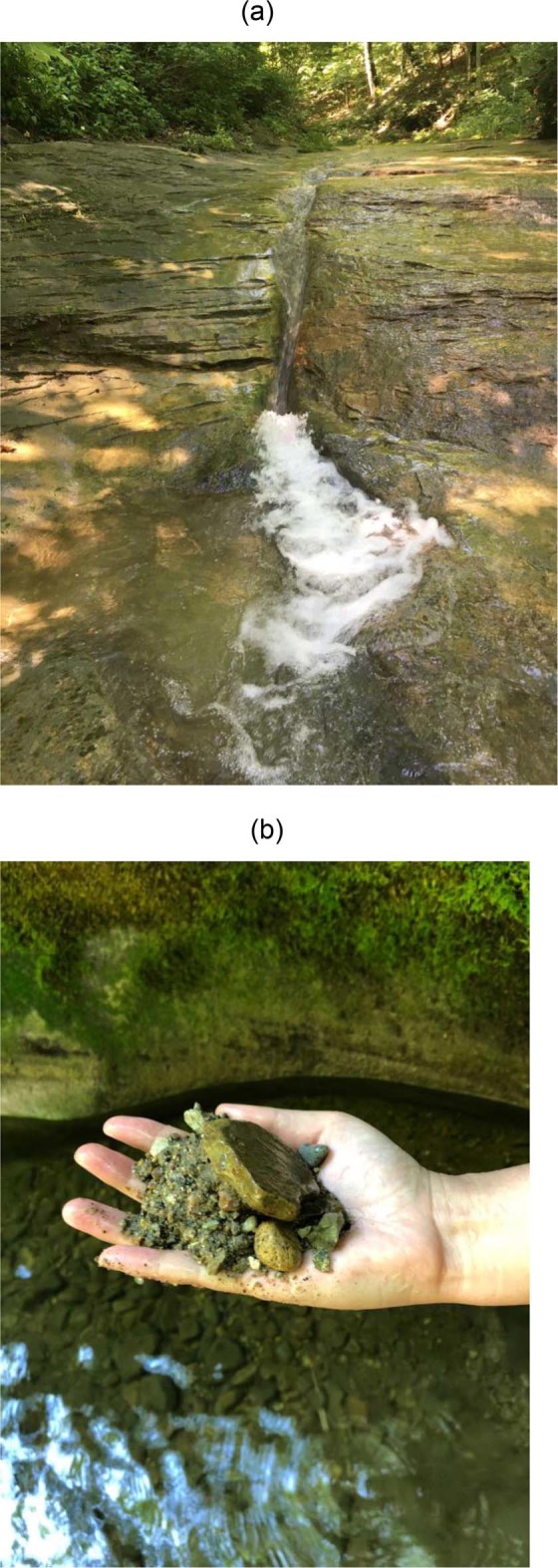
(**a**) Knickpoint with plunge pool carved into bedrock. Relief is ~1 m. (**b**) Alluvium excavated from an adjacent pothole. The site is at Fall Creek Gorge, Attica, Indiana, USA.

Bedrock rivers manifest a wide variety of morphologies^1,2^. Among these, knickpoints, both from low to high slope and high to low slope, are ubiquitous in bedrock-alluvial streams^3–5^. Figure 1 shows a steep reach of a small bedrock-alluvial stream followed by a plunge pool and a gentler reach. Alluvium is available in the plunge pool, as well as upstream and downstream. Here we address the following. (1) What are the conditions for the formation of plunge pools at breaks from high slope to low in mixed bedrock-alluvial streams^6,7^? (2) How does the long profile of the stream evolve near such slope breaks, with or without plunge pools? Does the profile tend to a shape that migrates upstream and vertically with constant speeds, without otherwise changing form (permanent form)^8,9^? Or does the coherence of the profile eventually break down?

Here we analyze the problem in a simplified way that casts light on knickpoint migration. Even though variation in channel width *B* in bedrock streams can be important^10^, we take it as constant. We replace the details of flood hydrographs^11–13^ with a simple flood intermittency such that the river floods for a fraction of time. While there are several mechanisms for bedrock incision^14–20^ here we consider abrasion due to saltating clasts^11^. We consider drilling effects^6,7^ only in the simple and incomplete but useful way that is embedded in the Macro-Roughness Saltation Abrasion Alluviation (MRSAA) model^21–23^. This method allows dynamic routing of alluvium and concomitant incision of a bedrock-alluvial river. Our model is 1D, and so cannot capture alternate bars migrating over bedrock^24^. In addition, we consider only a single, constant grain size *D*, and a constant uplift rate *υ*. Even with these restrictions, a rich pattern of behavior can be captured. Specifically, we illustrate the role of the Froude number ***Fr*** in plunge pool formation.

We use the MRSAA model^22,23^ to capture hydro-morphodynamics, but we model the flow using the 1D unsteady shallow water equations rather than the normal flow approximation used previously. The key advantage of the unsteady shallow water formulation is that it automatically captures hydraulic jumps. Let *U* = flow velocity, *H* = flow depth and *g* = gravitational acceleration: the Froude number is then given as ***Fr*** = *U*/*(gH)*^1/2^. Hydraulic jumps are manifested as jumps in ***Fr*** from above unity to below. We show that hydraulic jumps can mediate plunge pool formation.

The governing equations are given in Methods. Here we summarize them briefly. We use the 1D unsteady shallow water equations to compute the flow over a given bed. This bed may have vanishing to complete areal cover of alluvium, with complete cover and a corresponding cover fraction *p* of unity attained when the cover thickness *η*_*a*_ exceeds a value near the macro-roughness height *L*_*mr*_ of the bedrock itself. Alluvial morphodynamics is computed with an Exner equation modified to allow partial cover. The time rate of change of elevation *η*_*b*_ of the base of the bedrock is computed in terms of an uplift rate *υ* and an abrasionally driven incision rate that depends on an erosion coefficient *β*^23^.

Based on earlier work^22,23^, some parameters have been set to convenient values and held constant: *D* = 20 mm; *L*_*mr*_ = 1 m; *β* = 0.05 km^−1^; flood intermittency *I* = 0.05; Chezy resistance coefficient *Cz* = 10; and uplift rate *υ* = 5 mm/yr. Reach length *L* is set at 1000 m. As shown in the Table 1, water discharge per unit width *q*_*w*_ = 3 m^2^/s for all but one run. In 6 runs (Runs 3–8) the initial bed slope *S*_*bi*_ in the upstream half (*S*_*bi*_ (up)) of the reach is higher than the value for the downstream half (*S*_*bi*_ (dn)); in 2 runs (Run 1 and Run 2) they are equal, and in 1 run (Run 9) *S*_*bi*_ is higher downstream than upstream.

**Table 1:** Key parameters for numerical experiments.

Run	*q*_*w*_ m^2^/s	*q*_*af*_ m^2^/s	*S*_*bi*_ (up)	*S*_*bi*_ (dn)	*q*_*aci*_ (up) m^2^/s	*q*_*aci*_ (dn) m^2^/s	*Fr*_*i*_ (up)	*Fr*_*i*_ (dn)	*S*_*bss*_ (dn)	*Fr*_*ss*_
1	3	0.0523	0.15	0.15	0.105	0.105	3.87	3.87	0.075	2.78
2	3	0.00261	0.15	0.15	0.105	0.105	3.87	3.87	0.0055	0.75
3	3	0.000834	0.008	0.004	0.0042	0.0017	0.89	0.63	0.0027	0.52
4	3	0.000834	0.05	0.004	0.033	0.0017	2.23	0.63	0.0027	0.52
5	3	0.000834	0.075	0.004	0.051	0.0017	2.73	0.63	0.0027	0.52
6	3	0.000834	0.15	0.004	0.105	0.0017	3.87	0.63	0.0027	0.52
7	3	0.000834	0.5	0.004	0.36	0.0017	7.07	0.63	0.0027	0.52
8	3	0.000834	0.15	0.0005	0.105	0	3.87	0.22	0.0027	0.52
9	6	0.000834	0.004	0.15	0.004	0.21	0.63	3.87	0.0024	0.48

Here *q*_*w*_ = water discharge per unit width; *q*_*af*_  = volume feed rate of alluvium per unit width; *S*_*bi*_ (up) = upstream initial bed slope; *S*_*bi*_ (dn) = downstream initial bed slope; *q*_*aci*_ (up) = upstream initial capacity transport rate of alluvium; *q*_*ac*_ (dn) = downstream initial capacity transport rate of alluvium; ***Fr***_*i*_ (up) = initial upstream Froude number; ***Fr***_*i*_ (dn) = initial downstream Froude number; *S*_*bss*_ = final steady state bed slope; and ***Fr***_*ss*_ = final steady state Froude number.

Note: in the case of Run 8, the initial bed slope is so low (0.0005) that it forces the initial value of capacity bedload transport rate *q*_*ac*_ to vanish.

**Figure 2:**
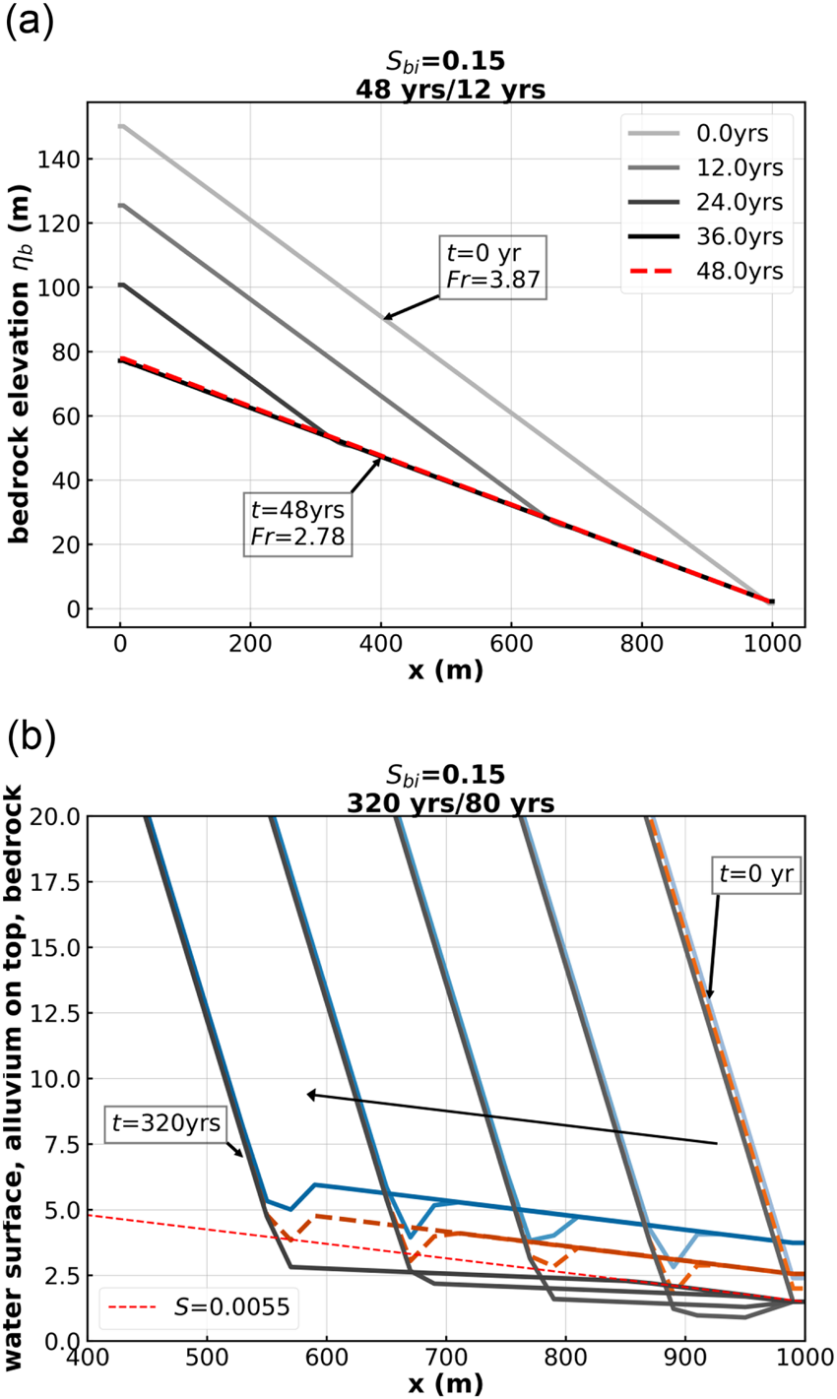
(**a**) Run 1 of the Table 1. (**b**) Run 2 of the Table 1. In (**a**), the lines with shading varying gradually from light to dark gray are bedrock profiles every 12 years, and the red dashed line is the bedrock elevation at the 48^th^ year. In (**b**), the blue lines denote water surface elevation, the orange lines denote the top of the alluvium, and the gray lines denote the bottom of the bedrock.

Figure 2 shows two cases with upstream-migrating knickpoints, Run 1 and Run 2, that differ in only a single aspect: the sediment feed rate in Run 1 is 20 times that of Run 2 (Table 1). Both cases have a spatially constant initial slope of 0.15, and an initial Froude number of 3.87. These conditions are preserved upstream of the knickpoint as it retreats. Downstream of the knickpoint, the profile regrades to the slope corresponding to steady-state balance between uplift and incision. In the case of Run 1 (Fig. 2a), the relatively high feed rate results in a relatively steep steady-state bedrock slope of 0.075 and Froude number of 2.78. The flow transitions from Froude-supercritical to slightly less Froude-supercritical, and no hydraulic jump forms. The knickpoint consists simply of a migrating slope discontinuity.

In the case of Run 2 (Fig. 2b), however, the lower feed rate results in a much lower steady-state slope, and a subcritical steady-state Froude number of 0.75. The flow must undergo a hydraulic jump at the knickpoint. This jump has been imprinted on the alluvial bed in terms of a scour hole, but there is no local plunge pool excavated into the bedrock.

**Figure 3:**
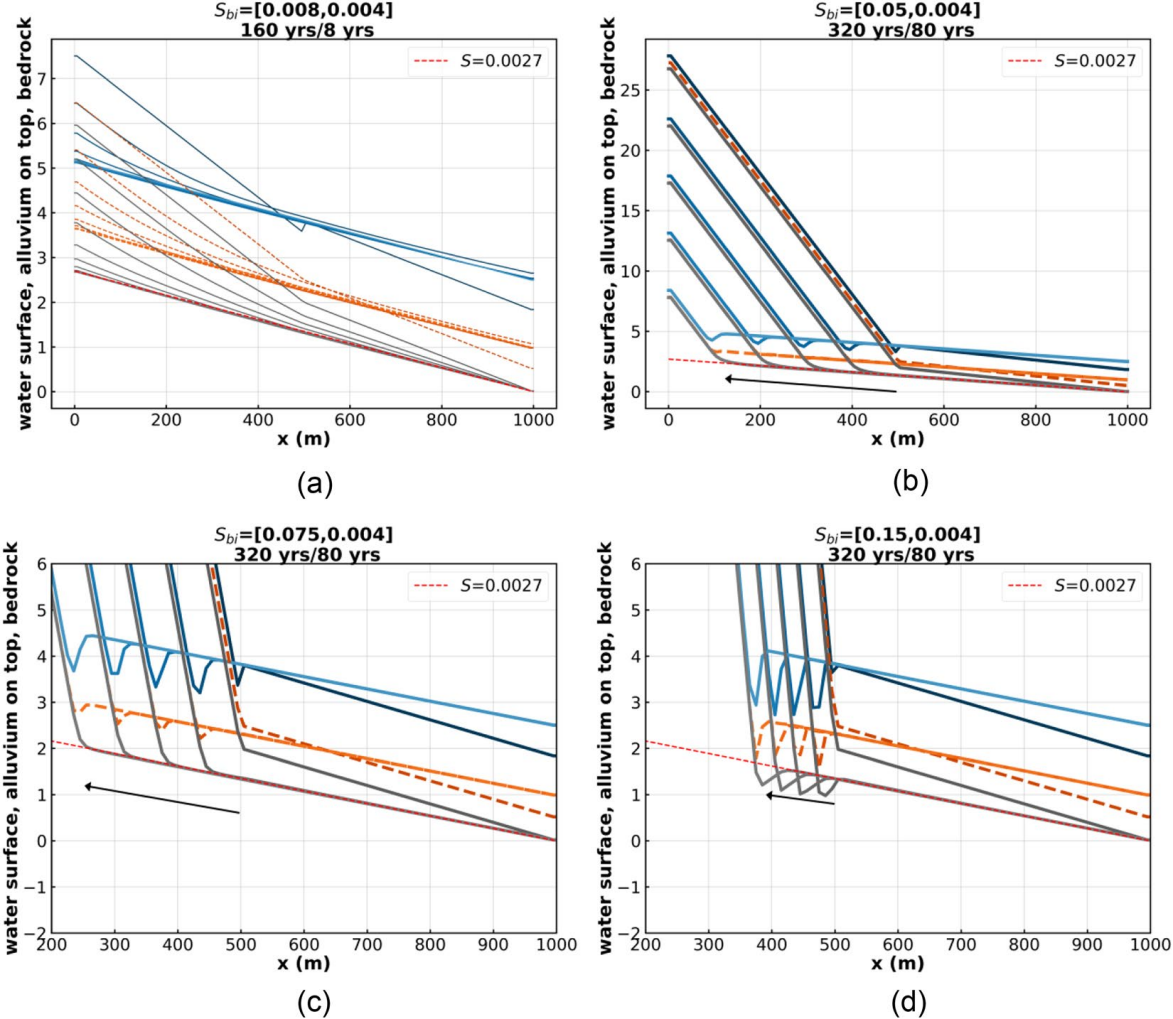
(**a**) Run 3 of the Table 1. (**b**) Run 4 of the Table 1. (**c**) Run 5 of the Table 1. (**d**) Run 6 of the Table 1. The blue lines denote water surface elevation, the orange lines denote the top of the alluvium, and the gray lines denote the bottom of the bedrock. Note the plunge pool in bedrock of Run 6.

Figure 3 shows four cases, Run 3, 4, 5 and 6, all with an initial mid-reach slope break, in which the only parameter that is varied is the initial bedrock slope for the upper half of the reach. It takes the values 0.008, 0.05, 0.075 and 0.15 for Run 3, 4, 5 and 6 correspondingly. The corresponding initial upstream Froude numbers are 0.89, 2.23, 2.73 and 3.87. The steady-state bedrock slope and Froude number are 0.0027 and 0.52 in all cases.

The sudden change in Froude number at the knickpoint increases in strength from Run 3 to Run 6. In the case of Run 3 (Fig. 3a), the flow is subcritical everywhere, and steady state is approached without a knickpoint. Indeed, runs under other conditions suggest that this behavior appears universal. In Run 4 (Fig. 3b), a Froude number transition from 2.23 to 0.52 gives a hydraulic jump but no alluvial scour hole or plunge pool. In Run 5 (Fig. 3c), a Froude number transition from 2.73 to 0.52 gives a hydraulic jump and an alluvial scour hole, but no plunge pool. In Run 6 (Fig. 3d), a Froude number transition from 3.87 to 0.52 gives a hydraulic jump, an alluvial scour hole and a plunge pool excavated into bedrock.

**Figure 4:**
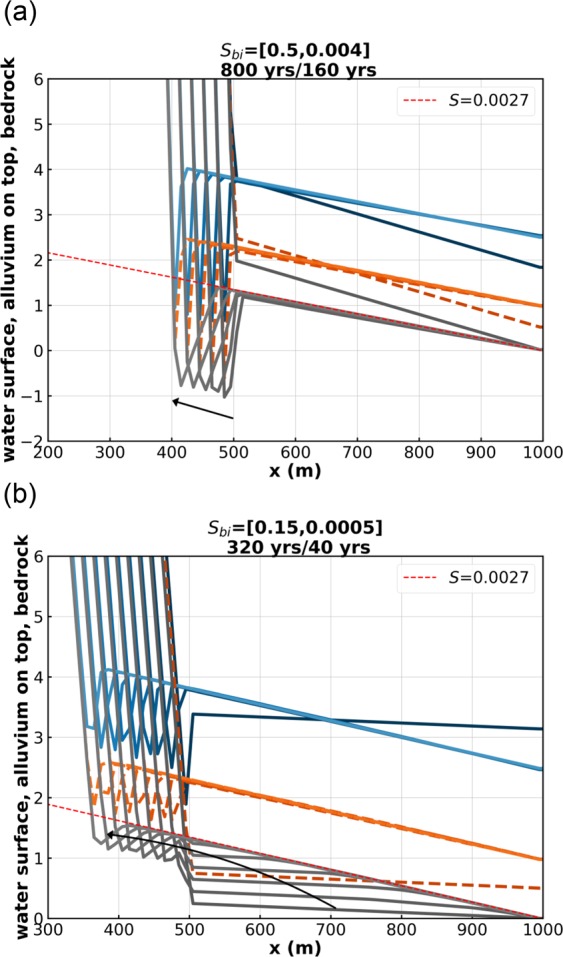
(**a**) Run 7 of the Table 1. (**b**) Run 8 of the Table 1. The blue lines denote water surface elevation, the orange lines denote the top of the alluvium, and the gray lines denote the bottom of the bedrock. Note the plunge pool in bedrock of Runs 7 and 8.

Figure 4 shows two cases, Runs 7 and 8, for which substantial local plunge pools form. Run 7 (Fig. 4a), which is a continuation of the series of Runs 3, 4, 5 and 6, has a Froude number transition from 7.07 to 0.52. The initial slope is 0.5, a value that is somewhat outside the bounds of the standard shallow water formulation, but this case is included to illustrate limiting behavior. With this sharp transition in Froude number, a plunge pool of about a meter in depth forms. Runs 4 to 7 illustrate the control the Froude number exerts on hydraulic jump, alluvial scour hole and plunge pool formation.

Run 8 of Fig. 4b is identical to that of Run 6 (Fig. 3d), except for the fact that the initial bed slope of the downstream half of the reach has been lowered from 0.004 to 0.0005. The initial downstream sediment transport rate is vanishing in this case. As a result, two knickpoints appear as the channel regrades to steady-state. There is a downstream knickpoint with no hydraulic jump, similar to the one in Fig. 2a, corresponding to a regrading to the steady-state slope of 0.0027, and an upstream one with a hydraulic jump, an alluvial scour hole, and a plunge pool.

Cases which show knickpoints with hydraulic jumps, i.e. Run 2 (Fig. 2b), Run 4 (Fig. 3b), Run 5 (Fig. 3c), Run 6 (Fig. 3d), Run 7 (Fig. 4a) and Run 8 (Fig. 4b) share a common feature. If allowed to migrate far enough upstream, they tend to a profile of constant form, which migrates upstream at constant speed *c*_*x*_ and in the vertical at constant speed *c*_*z*_ without changing shape^8,9^. This behavior is slightly obscured in e.g. Run 7 (Fig. 4a), because of oscillations associated with migration through the numerical grid. In the case of Run 4 (Fig. 3b), the migrating velocity in the x and z directions (*c*_*x*_, *c*_*z*_) = (−1.28 m/year, 0.0031 m/year); in the case of Run 6 (Fig. 3d), (*c*_*x*_, *c*_*z*_) = (−0.41 m/year, 0.0011 m/year); and in the case of Run 7 (Fig. 4a), (*c*_*x*_, *c*_*z*_) = (−0.12 m/year, 0.0004 m/year).

**Figure 5:**
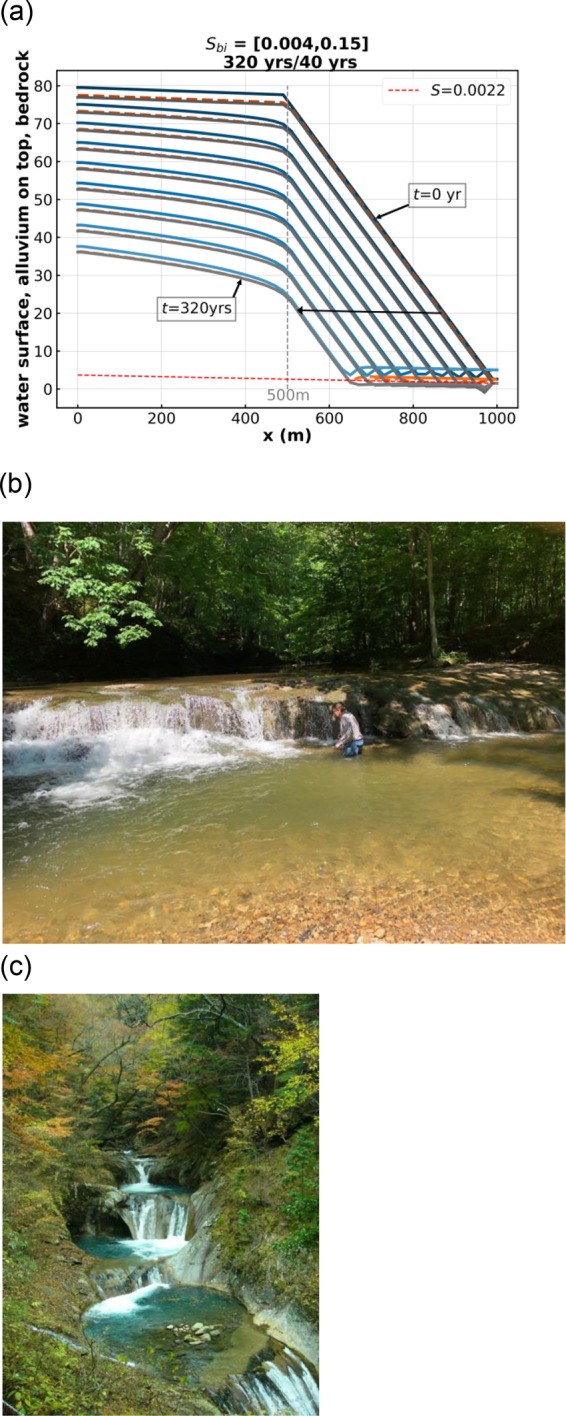
(**a**) Run 9 of the Table 1. The blue lines denote water surface elevation, the orange lines denote the top of the alluvium, and the gray lines denote the bottom of the bedrock. (**b**) Waterfall in Fall Creek Gorge, Attica, Indiana USA. Note the abundant alluvium downstream of the waterfall. The human provides the scale. The upper profile is downward concave toward the waterfall. (**c**) Series of plunge pools (cyclic steps) with partial fill of alluvium, Nanatsugama Godan no Taki, Yamanashi, Japan. Step height is 2–3 m.

A corresponding solution of permanent form is not obtained, however, when the initial imposed slope break is from low to high. In Run 9 (Fig. 5a), the ordering of the initial slopes is reversed as compared to Runs 3 to 8: the initial upstream slope is 0.004 (low) and the initial downstream slope is 0.15 (high). The initial Froude number is 0.63 upstream and 3.87 downstream, so a hydraulic jump does not occur. As can be seen from Fig. 5a, while regrading progresses from the downstream end, the initial break from low to high slope dissipates in place without migrating. In addition, the initial low upper slope devolves into a downward-concave profile, which eventually obliterates the slope break. Such a downward-concave upper profile can be seen in a bedrock stream in the field in Fig. 5b. The model given here does not, then, appear to be suitable for modeling the trains of plunge pools, partially filled in bedrock (cyclic steps) that are commonly seen in steep bedrock streams in nature (Fig. 5c). This may be due to the choice of input parameters^9^ or missing physical processes such as drilling^6^. In point of fact, the present model is able to describe drilling, but only in a crude form that fits into the 1D shallow water rubric.

## Discussion

The equation of momentum balance of the shallow water formulation used for the runs presented above has no angle correction for very steep slopes. In addition, the abrasion coefficient *β* is taken to be constant, rather than allowed to vary^25^. In Methods we outline the complete morphodynamic formulation, including the shallow water equations with and without an angle correction, and the MRSAA formulation with and without variable coefficient *β*. In the Supplementary Information we show results for the conditions corresponding to Run 4 and Run 5 of the Table 1, for case Sa) the original formulation (no angle correction and constant *β*), case Sb) (angle correction and constant *β*), case Sc) (no angle correction and variable *β*) and case Sd) (angle correction and variable *β*). Broadly similar results are obtained for all cases, but the case of variable *β* gives a deeper plunge pool into bedrock.

We summarize our model results as follows.

- Migrating knickpoints can form at transitions from high supercritical to low supercritical flow, but these are slope discontinuities with no alluvial scour hole or plunge pool in bedrock.
- Initial slope breaks dissipate without forming knickpoints when the flow is everywhere subcritical.
- Slope breaks from supercritical to subcritical flow form knickpoints with hydraulic jumps that can eventually migrate upstream and vertically while maintaining permanent form. When the difference in Froude number is sufficiently large, the knickpoint includes an alluvial scour hole and a plunge pool excavated into bedrock.
- Transitions from low-supercritical flow to high supercritical flow tend to dissipate without forming a knickpoint.

We emphasize three final points here. 1. The vertical scale for the numerical runs extends over 100’s to 1000 m (Figs. 2–5, S1 and S2) whereas the vertical scale apparent in the field expression of Fig. 1 is a few meters. We resolve this in Fig. S3 of the Supplementary Information, where we show that the plunge pool itself has a scale of meters, regardless of the horizontal and vertical scale of the study reach itself. 2. Knickpoints with plunge pools are ubiquitous features of mountain streams^3–5,13,14^. Yet only a few papers have concentrated on the theoretical or experimental modeling of plunge pools at knickpoints^6,7,9,18,26^. We believe that our shallow water MRSAA model implementation will have wide applicability to the study of both the 1D and 2D manifestations of bedrock plunge pools. 3. We show in the Supplementary Information that the basic form of the plunge pools modeled here is retained when water supply is doubled compared to Run 6, and when the constant water supply and sediment feed rates of Run 6 are replaced with a hydrograph and sedimentograph.

Our results as a whole invite further experimental and field research^6,7,26^.

## Methods

### Governing equations

Here we model the flow using the equations of water mass and momentum conservation constituting the 1D unsteady shallow water equations:

1 $$\frac{{\rm{\partial }}H}{{\rm{\partial }}t}+\frac{{\rm{\partial }}}{{\rm{\partial }}x}(UH)=0,$$

2 $$\frac{\partial }{\partial t}(UH)+\frac{\partial }{\partial x}({U}^{2}H)=-\,gH\frac{\partial H}{\partial x}+gHS-{C}_{f}{U}^{2},$$

where *x* = streamwise distance*, t* = time, *H* = depth, *U* = flow velocity, *g* = gravitational acceleration, *C*_*f*_ = dimensionless bed friction coefficient, and *S* = slope of the bed surface. In the present work, *C*_*f*_ is a specified constant = (*Cz*)^−2^ where *Cz* is a dimensionless Chezy resistance coefficient. Bed surface slope is given as

3 $$S=-\,\frac{\partial }{\partial x}({\eta }_{b}+{\eta }_{a}),$$

where *η*_*b*_ = elevation to the base of the bedrock and *η*_*a*_ = mean thickness of alluvial cover. Equation (2) should be corrected for slope angles in excess of 0.39 radians (22.3°) as follows:

4 $$\frac{\partial }{\partial t}(UH)+\frac{\partial }{\partial x}({U}^{2}H)=-\,\frac{1}{2}g\frac{\partial ({H}^{2}\,\cos \,\theta )}{\partial x}+gH\,\sin \,\theta -{C}_{f}{U}^{2}.$$

Alluvial and bedrock morphodynamics are formulated as follows. The bedrock is assumed to have a partial cover of alluvium. Let *p* denote the areal fraction of bedrock surface that is covered with alluvium. Then the equation of conservation of alluvium is

5 $$(1-\lambda )p\frac{\partial {\eta }_{a}}{\partial t}=-\,I\frac{\partial }{\partial x}({q}_{ac}{p}_{a}).$$

Here *λ* is porosity of the alluvium, *q*_*ac*_ is the capacity volume transport rate per unit width of alluvium, *I* is the flood intermittency (*I* = 1 for continuous flood flow) and *p*_*a*_ is an adjusted cover fraction for alluvium available for dislodgement. The relations for *p* and *p*_*a*_ are

6 $$p=\{\begin{array}{cc}{p}_{l}+({p}_{h}-{p}_{l})\frac{{\eta }_{a}}{{L}_{mr}}, & 0\le \frac{{\eta }_{a}}{{L}_{mr}}\le \frac{1-{p}_{l}}{{p}_{h}-{p}_{l}}\\ 1, & \frac{{\eta }_{a}}{{L}_{mr}} > \frac{1-{p}_{l}}{{p}_{h}-{p}_{l}},\end{array}$$

7 $${p}_{a}=\frac{p-{p}_{l}}{1-{p}_{l}},$$

where *L*_*mr*_ denotes an intrinsic macro-roughness of the bedrock, taken as constant here, and *p*_*h*_ and *p*_*l*_ take the respective reference values 0.95 and 0.05.

The equation of conservation of bedrock is

8 $$\frac{\partial {\eta }_{b}}{\partial t}=\upsilon -I\beta {q}_{ac}{p}_{a}(1-{p}_{a}),$$

where *υ* is a rock uplift rate and *β* is a coefficient of bedrock abrasion. The latter is taken as constant in the main text, but is allowed to vary below^25^.

The alluvium is assumed to be gravel with size *D* and submerged specific gravity *R* (1.65 for quartz); the capacity bedload transport relation used here is the modified form of Wong and Parker^27^ of the relation of Meyer-Peter and Müller (1948) as follows:

9 $${q}_{ac}=4\sqrt{RgD}\,D{({\tau }^{\ast }-{\tau }_{c}^{\ast })}^{3/2},$$

where the Shields stress τ* is given as

10 $${\tau }^{\ast }=\frac{{C}_{f}{U}^{2}}{RgD},$$

and $${\tau }_{c}^{\ast }$$ = 0.0495^27^.

The relation for variable abrasion coefficient *β* can be cast in the following form^22,25^

11 $$\beta ={\beta }_{ref}\frac{{(\frac{{\tau }^{\ast }}{{\tau }_{c}^{\ast }}-1)}^{-\frac{1}{2}}{[1-{(\frac{{u}_{\ast }}{{v}_{s}})}^{2}]}^{\frac{3}{2}}}{{(\frac{{\tau }_{ref}^{\ast }}{{\tau }_{c}^{\ast }}-1)}^{-\frac{1}{2}}{[1-{(\frac{{u}_{\ast ,ref}}{{v}_{s}})}^{2}]}^{\frac{3}{2}}}={\beta }_{ref}\frac{{(\frac{{\tau }^{\ast }}{{\tau }_{c}^{\ast }}-1)}^{-\frac{1}{2}}{[1-\frac{{\tau }^{\ast }}{{{R}_{f}}^{2}}]}^{\frac{3}{2}}}{{(\frac{{\tau }_{ref}^{\ast }}{{\tau }_{c}^{\ast }}-1)}^{-\frac{1}{2}}{[1-\frac{{\tau }_{ref}^{\ast }}{{{R}_{f}}^{2}}]}^{\frac{3}{2}}}.$$

Here *β*_*ref*_  is a reference value of *β* prevailing when the Shields number τ^*^ is equal to the reference value *τ*_ref_^*^ and the shear velocity u_*_ = (*C*_*f*_)^1/2^*U* is equal to the corresponding reference value ${u}_{\ast ,{ref}}$. Here we take *β*_*ref*_ = 0.05 km^−1^. The parameter *v*_*s*_ denotes the fall velocity of the sediment, and *R*_*f*_  = *v*_*s*_/(*RgD*)^1/2^.

### Numerical methods

The governing equations, namely, the unsteady shallow water equations (equations (1) and (2)), the equation of conservation of alluvium (equation (5)), and the equation of conservation of bedrock (equation (8)), are discretized in a staggered grid system. Scalar values such as water depth, alluvial thickness, and bedrock elevation are specified at the center of each cell, and vector values such as flow velocity and bedload flux are specified at the sides of the cell. We use a 1^st^ order upwind scheme to discretize the advection terms of the governing equation (second term of left hand side of equations (1) and (2), and the term of right hand side of equation (5)) for simplicity and computational stability. A 1^st^ order explicit scheme is used to discretize the time derivative of the governing equations. For discretization of the unsteady terms, we use three different time step lengths *Δt* in the numerical solution: a very short step *Δt*_*h*_ (10^−2^∼10^−1^ s) for hydrodynamics, a much longer step *Δt*_*a*_ (10^−6^∼10^−5^ yrs) for alluvial morphodynamics, and an even longer time step *Δt*_*b*_ (10^−5^∼10^−4^ yrs) for bedrock morphodynamics. These are scaled as fractions of the following respective time scales: *H*^2^*/q*_*w*_, *H*^2^*/q*_*ac*_ and *H/*(*βq*_*ac*_), where representative values are used for each of these parameters.

## Supplementary information

Supplementary Material
