# Supplementary material for: Bedrock-alluvial streams with knickpoint and plunge pool that migrate upstream with permanent form
Source: Sci Rep. 2019 Apr 16;9:6176. doi: 10.1038/s41598-019-42389-2 (PMC6467923; doi:10.1038/s41598-019-42389-2)
Supplement: Supplementary file 1 — Supplementary Material [file 41598_2019_42389_MOESM1_ESM.pdf]

Bedrock-alluvial streams with knickpoint and plunge pool that migrate upstream with permanent form

Li Zhang[1,2*,3], Toshiki Iwasaki[4], Tiejian Li[2,3], Xudong Fu[2], Guangqian Wang[2,3] and Gary Parker[1,5]

[1]Department of Civil & Environmental Engineering, University of Illinois Urbana-Champaign, Urbana, 61801 USA,

[2] State Key Laboratory of Hydroscience and Engineering, Tsinghua University, Beijing, 100086 China,

[3] School of Water Resources and Electric Power, Qinghai University, Xining, 810016 China,

[4]Civil Engineering Research Institute for Cold Regions, Sapporo, 062-8602 Japan,

[5]Department of Geology, University of Illinois Urbana-Champaign, Urbana, 61801 USA.

*e-mail: (lizhangpig@gmail.com).

SUPPLEMENTARY INFORMATION

Calculations Including Slope Angle Correction and Variable Abrasion Coefficient

In order to test the role of the slope angle correction and variable abrasion coefficient, we performed four extra runs based on Run 4 of Table 1 (Fig. 3b), and four extra runs based on Run 5 of Table 1 (Fig. 3c). In the case of Run 4, Figure S1a shows results with no angle correction and constant β; the results are identical to Run 4 (Fig. 3b). In Fig. S1b, the angle correction corresponding to equation (4) has been implemented. In Fig. S1c, variable β is implemented according to equation (11), with β_{ref} = 0.05 km^{-1} and τ^*_{ref} computed from normal flow conditions over the upper half of the initial bed (S_{bi} (up) = 0.05). In Fig. S1d, both the angle correction and variable β have been implemented.

Bedrock profiles for the four cases pertaining to Run 4 are shown in Fig. S1. In the macroscopic view of Fig. S1, there is little difference between the four cases. It is seen from the insets in Figs. S1a~d, however, that the case of variable β gives a steepened slope just upstream of the slope break, and also predicts a small plunge pool that is not captured by the case of constant β until the difference in Froude number is more extreme.

The corresponding extra runs for Run 5 are shown in Fig. S2. The same behavior is found as in Fig. S1. At the initial upstream slope angles considered in the cases of Figs. S1 and S2 (2.9° and 4.3°) respectively, the angle correction is hardly felt at all. This would, of course, not be the case for near-vertical waterfalls. The case of variable β leads to the onset of plunge pool formation at a smaller difference in initial upstream and steady-state Froude numbers than in the case of constant β.

The formulation for variable β is more physically based and might be the preferred option for modeling. As initial upstream slope increases beyond 0.15, however, our model tends to become numerically unstable. This might be associated with the term $(1 - \tau^*_{ref}/R_f^2)$ in equation (11), which becomes vanishing at sufficiently steep slope.

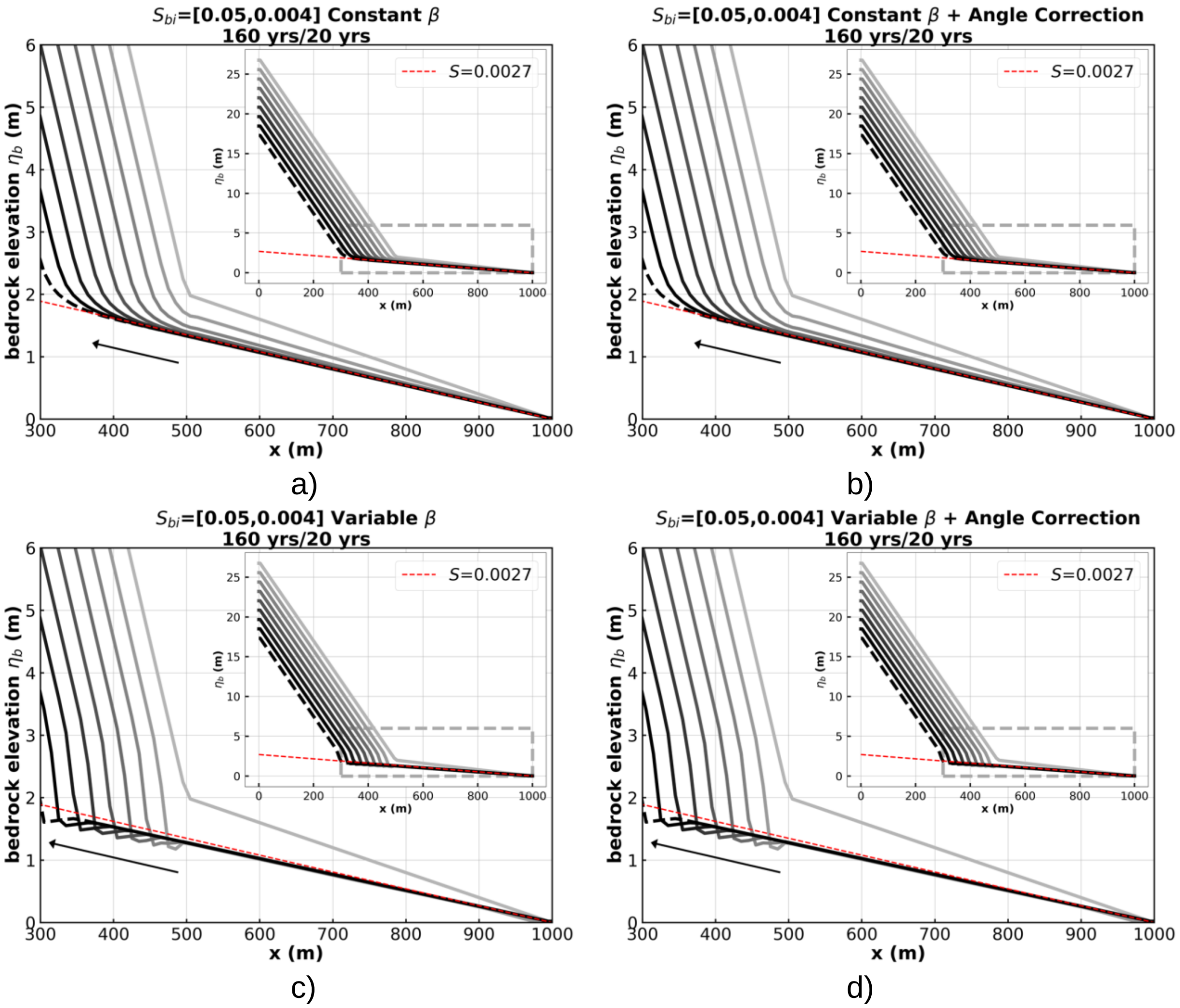

Figure S1. a) Run 4, constant β and no angle correction: identical to Fig. 3b; b) Run 4, constant β with slope angle correction; c) Run 4, variable β; d) Run 4, variable β and slope angle correction.

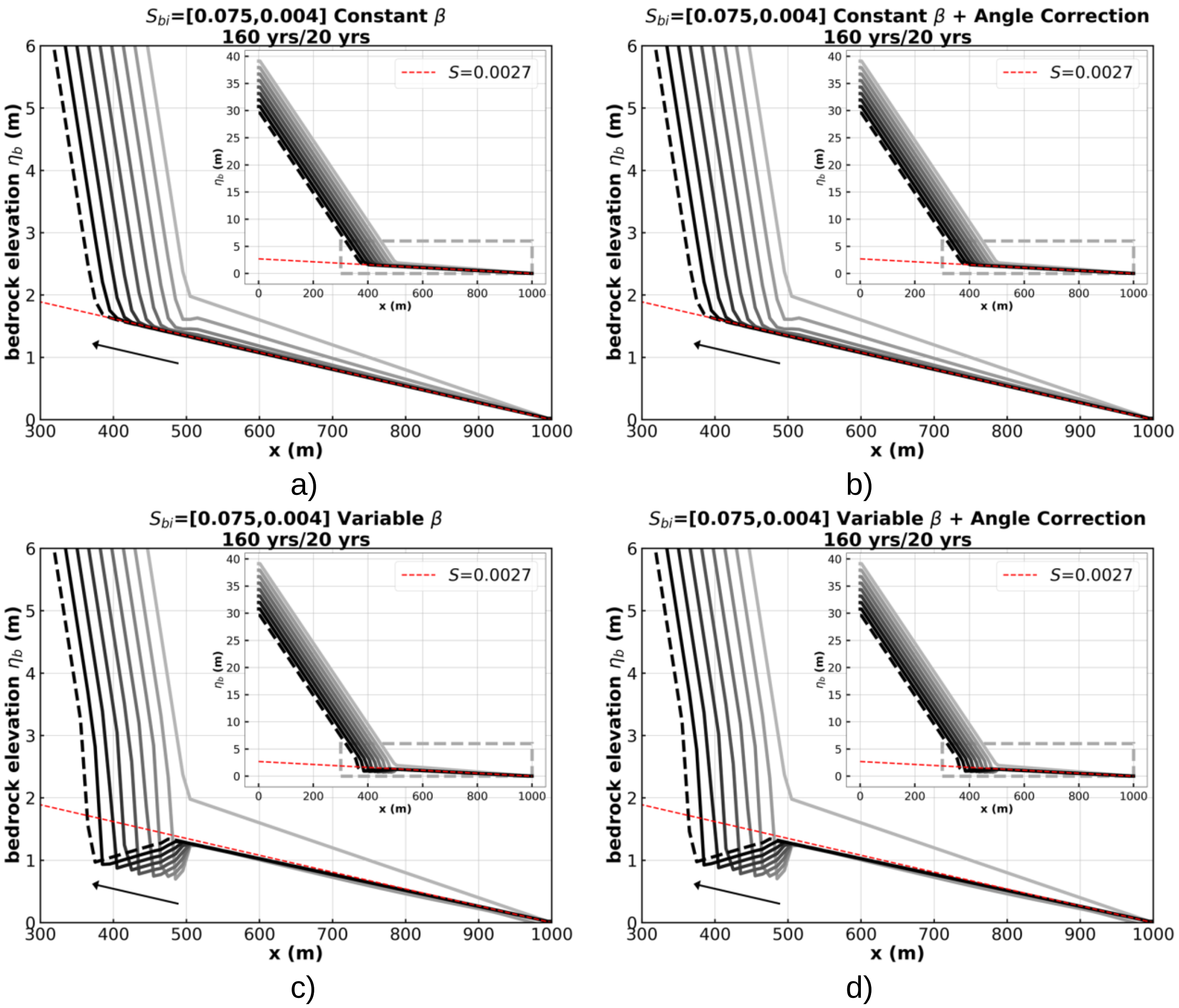

Figure S2. a) Run 5, constant β and no angle correction: identical to Fig. 3c; b) Run 5, constant β with slope angle correction; c) Run 5, variable β; d) Run 5, variable β and slope angle correction.

Effect of Scale

For the cases of high to low slope transition, the calculations above are based on a reach length of 1000 m and vertical ranges of 7-250 m (Figs. 2, 3, 4, S1 and S2). Yet our main field example, shown in Fig. 1, involves a scale of m in the vertical and 10's of m in the horizontal. We demonstrate in Fig. S3 below that there is no inconsistency here. In the figure, the depth of the bedrock plunge pool is around 2 m, of the same order of magnitude as that seen in Fig. 1a.

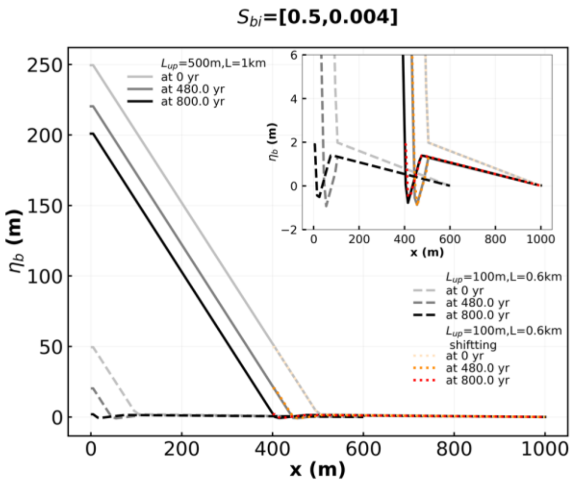

Figure S3. Results for two sub-cases of Run 7 (Fig. 4a) to illustrate the structure of the bedrock surface far from the plunge pool (bottom left plots; vertical scale ~250 m) and in the vicinity of the plunge pool (upper right plots; vertical scale ~ 8 m). The initial slope breaks are at *x* = 500 m (Run 7) and *x* = 100 m. It is seen that the position of the slope break has no effect on the evolving structure. Plunge pool depth is ~ 2 m.

Effect of Variation in Water and Sediment Supply Rate

We use as a basis for calculations with variable water and sediment supply conditions Run 6, which has constant water and sediment supply rate. In Run 10 below, the conditions are identical to those of Run 6, except that q_w has been increased from the constant value 3 m^2/s to the constant value 6 m^2/s. In Run 11 below, the constant supply rates of water q_w and sediment q_{feed} have been replaced with the cycling hydrograph and sedimentograph shown in Figs. S4a and S4b. The results of the calculations are shown in Fig. S5. Increasing q_w alone results in a plunge pool with a larger relief from pool bottom to top of hydraulic jump, but the same structure as that of Run 6. The results for Run 11 are nearly identical to Run 6.

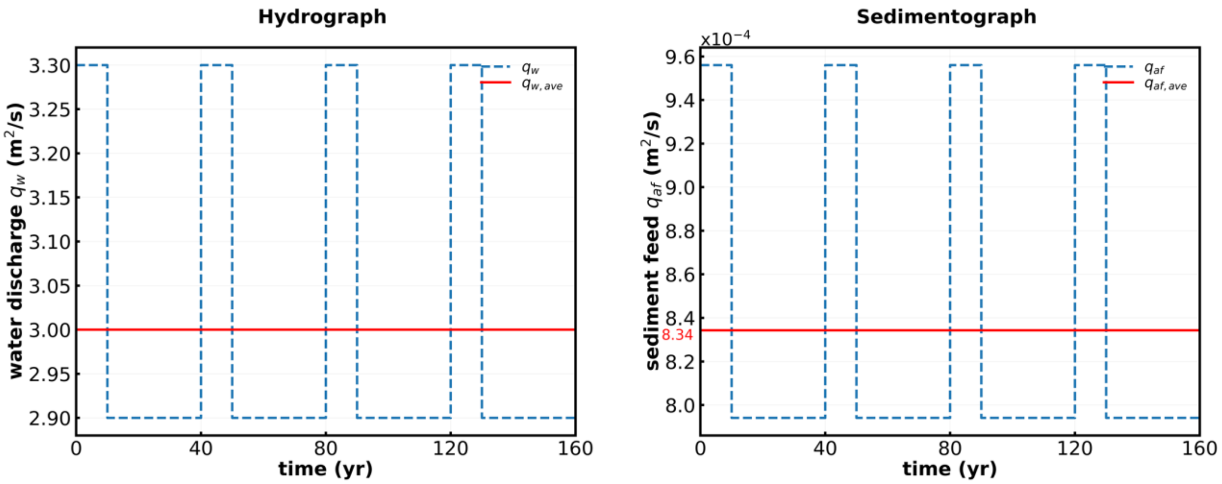

a) b)

Figure S4. a) Time-varying hydrograph used as upstream conditions for Run 11 explained below. The time-averaged value of $q_{w,ave}$ = 3 m^2/s, i.e. the same as the constant value for Run 6; b) Time-varying sedimentograph used as upstream conditions for Run 11 explained below. The time-averaged value of $q_{af,ave}$ = 0.000834 m^2/s is the same as the constant value for Run 6.

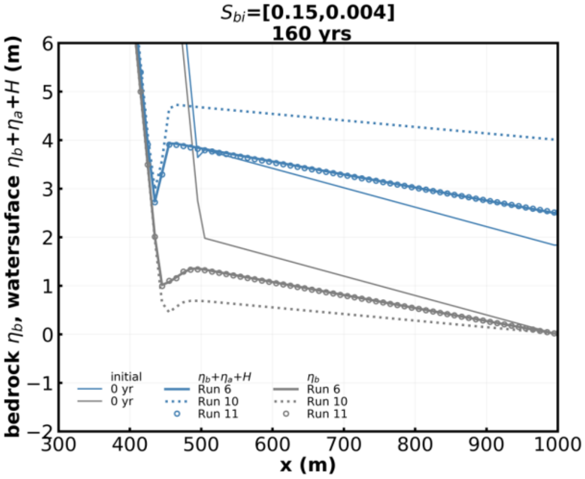

Figure S5. This figure repeats Fig. 3d pertaining to Run 6 (constant water and sediment supply rate). It also shows results for Run 10, which has the same conditions except that the water supply rate has been doubled, and for Run 11, which has the same conditions as Run 6 except that the water and sediment supply rate have been varied cyclically (hydrograph and sedimentograph).
